# Factors influencing home blood pressure monitor ownership in a large clinical trial

**DOI:** 10.1038/s41371-021-00511-w

**Published:** 2021-03-02

**Authors:** Thineskrishna Anbarasan, Amy Rogers, David A. Rorie, J. W. Kerr Grieve, Robert W. V. Flynn, Thomas M. MacDonald, Isla S. Mackenzie

**Affiliations:** Medicines Monitoring Unit (MEMO Research), Division of Molecular and Clinical Medicine, University of Dundee, Dundee, UK

**Keywords:** Health care, Clinical trials, Hypertension

## Abstract

Home blood pressure monitor (HBPM) ownership prevalence and the factors that influence it are unclear. This study aimed to investigate factors associated with HBPM ownership among participants in the Treatment in Morning versus Evening (TIME) hypertension study. This study is a sub-analysis of the TIME study, a randomised trial investigating the effect of day-time versus night-time dosing of antihypertensive medication on cardiovascular outcomes in adults with hypertension. As part of the TIME study online registration process, participants were asked to indicate whether they owned an HBPM. A multivariable logistic regression model was constructed to determine factors associated with HBPM ownership. Of 21,104 randomised participants, 11,434 (54.2%) reported owning an HBPM. The mean age of all participants at enrolment was 67.7 ± 9.3 years, 12,134 (57.5%) were male, and 8892 (42.1%) reported a current or previous history of smoking. Factors associated with an increased likelihood of reporting HBPM owned include being male (OR:1.47; 95% CI 1.39–1.56) or residing in a less deprived socioeconomic region (IMD Decile 6–10) (OR:1.31; 95% CI 1.23–1.40). Participants with a history of diabetes mellitus (OR:0.74; 95% CI:0.64–0.86) or current smokers, compared to non-smokers, (OR:0.71; 95% CI:0.62–0.82) were less likely to report owning an HBPM. This study has identified important patient factors influencing HBPM ownership. Further qualitative research would be valuable to identify and explore potential patient-level barriers to engagement with self-monitoring of blood pressure.

## Introduction

Home blood pressure monitoring, combined with either self or clinician-led titration of antihypertensive medication, is increasingly recognised as an effective strategy to improve blood pressure (BP) control in patients with hypertension [1, 2]. Home blood pressure monitor (HBPM) measurements, in comparison to clinic BP measurements, have been reported to be more reproducible [3] and are more predictive of cardiovascular mortality [4, 5]. Other advantages include greater convenience, the ability to make multiple measurements over a prolonged period, avoidance of white coat effect, and improved patient engagement with BP management [6–8].

The prevalence of HBPM use is increasing, with estimates that between a third and two-thirds of patients with hypertension in the UK are using HBPMs for self-monitoring [9, 10]. Global estimates for the prevalence of HBPM self-monitoring are between 30 and 70% [11]. Meta-analyses have shown reduced systolic and diastolic BP and overall cost savings when an HBPM-based intervention is used in conjunction with multidisciplinary care and education for patients with hypertension [12, 13].

Identifying factors that may motivate or hinder patients from owning HBPMs for self-monitoring may allow for targeted interventions. Fear of disease progression, curiosity, and clinician advice have been identified as motivators to own and use HBPMs [14]. Additionally, patient-level demographic and medical factors (e.g. past medical and family history) can influence ownership of medical devices for self-monitoring. For example, in patients with a history of stroke, the physical task of operating HBPMs was identified as a significant deterrent for using HBPMs [15].

This study aimed to investigate patient-level demographic, medical, and socioeconomic factors associated with HBPM ownership in participants of the Treatment In Morning versus Evening (TIME) study [16]. The TIME study is a randomised trial investigating the effect of day-time versus night-time dosing of antihypertensive medication on cardiovascular outcomes in adults with hypertension.

## Methods

The TIME study is a prospective, randomised, open-label, blinded end-point design remote decentralised clinical trial investigating the effect of day-time versus night-time dosing of antihypertensive medication on cardiovascular outcomes in 21,104 participants with hypertension [16]. A secure study-specific website is used to collect information directly from participants. Patients above the age of 18, diagnosed with hypertension and prescribed at least one antihypertensive medication to be taken once daily and with a valid email address were eligible to participate in the TIME study. Patients taking twice daily antihypertensive therapy, working shift patterns or involved in another clinical trial within the last 3 months were deemed ineligible to participate. Eligible patients enrolled in the TIME study via a secure web platform (https://www.timestudy.co.uk/). Invitations to participate in the TIME study were sent to patients from primary and secondary care practices across the UK and from databases of individuals who had previously indicated an interest in participating in research studies. Prospective participants were invited to complete an online form which evaluated study eligibility based on the inclusion and exclusion criteria. Eligible participants were then required to complete an online consent and registration process before randomisation.

The TIME study is a registered clinical trial (EudraCT 2011-001968-21, ISRCTN18157641) with ethical approval (East of Scotland Research Ethics Service 11/AL/0309).

### Data collection

An online form, accessible via the TIME study website, was used to collect relevant baseline information from participants regarding demographics and medical history. A combination of check-boxes and drop-down menus were provided for participants to indicate their responses for variables (summarised in Table 1). The primary outcome measurement (HBPM ownership status) was elicited as a binary variable with two options (yes/no). All collected data were held in a secure data server as part of the TIME study and anonymised before analysis.

**Table 1:** Summary of relevant variables collected from the TIME study online form.

Relevant variables collected in online form	Remarks
*Selection from down menu or check-box*	
Date of birth	–
Gender	Options: Male, female
Smoking status	Options: current smoker; ex-smoker; non-smoker, unknown
Co-morbidities	Options: detailed in Methods
Prescription of lipid lowering medication	Options: yes; no, unknown
Number of anti-hypertensive medications	Numeric options provided
Parents history of hypertension	Options: yes; no, unknown
Siblings/children history of hypertension	Options: yes; no, unknown
CV event^a^ in 1st degree relative age <60	Options: yes; no, unknown
CV event in 2nd degree relative age <50	Options: yes; no, unknown
Ownership of HBPM	Options: yes; no
*Free-text/string entry*	
Height (m)	BMI derived from height and weight.
Weight (kg)	
Systolic blood pressure (mmHg)	Baseline self-reported blood pressure reading.
Diastolic blood pressure (mmHg)	
Residential postal-code^b^	Individual index of multiple deprivation (IMD) decile scores were derived from their residential postal codes using the respective national guidance on deprivation scoring:
	—Scotland (https://www.gov.scot/Topics/Statistics/SIMD)
	—England (https://imd-by-postcode.opendatacommunities.org/)
	—Wales (https://gov.wales/statistics-and-research/welsh-index-multiple-deprivation)
	—Northern Ireland (https://www.nisra.gov.uk/statistics/deprivation/northern-ireland-multiple-deprivation-measure-2017-nimdm2017)

^a^Cardiovascular (CV) event refers to stroke, transient ischaemic attack or myocardial infarction.

^b^Residential postal codes were held in the secured TIME study database and only derived decile scores were extracted for analysis in this study.

The baseline form elicited personal history of the following health conditions: diabetes mellitus, angina, chronic obstructive pulmonary disease, myocardial infarction, impaired kidney function, peripheral vascular disease, arthritis, stroke and transient ischaemic attack (TIA). A composite variable, labelled comorbidity burden was derived to evaluate the number of comorbidities additional to hypertension that participants reported. Participants were classified as follows: no additional comorbidity, 1–2 comorbidities and ≥3 comorbidities.

Recruitment to the TIME study included participants from across the UK. Individual index of multiple deprivation (IMD) decile scores were derived from their residential postal codes using the respective national guidance on deprivation scoring detailed in Table 1. Socioeconomic deprivation scores for each participant were collected as a single variable, labelled IMD decile score. Subsequently, participants were stratified into two socioeconomic groups for analysis; more deprived (IMD decile 1–5) or less deprived (IMD decile 6–10).

Recruitment to the TIME study began in 2011 and was completed in 2017. Over this period, public marketing of HBPMs increased and the awareness of self-measured BP in the UK, improved significantly [17]. Hence, to consider the influence of the increased awareness with time on HBPM ownership, a variable was derived to classify participants based on their year of registration to the TIME study (2011–2013, 2014–2015 and 2016–2017).

### Statistical analysis

Potential predictors of HBPM ownership were included in a multivariable logistic regression model. These included: age, gender, smoking status, BMI, individual comorbidities, comorbidity burden, prescription of lipid-lowering drugs, family history of hypertension and cardiovascular events, number of antihypertensive medications, socioeconomic deprivation, country of residence at study enrolment and year of registration to TIME study. Country of residence was included in multivariable regression analysis to adjust for country-specific differences in IMD decile scores. Results of the logistic regression model are presented as odds ratios with associated 95% confidence intervals. Further analysis was performed to examine possible interactions between gender and co-morbidities [18].

To adjust for patient error in data reporting, for variables captured via text-entry (BMI, calculated from height and weight; systolic and diastolic BP), outlying data points for these variables (within 0.5% of either end of the distribution) were excluded from analysis. Before excluding extreme data points, variables were checked for normality of distribution using the Shapiro Wilk test. In total, the number of excluded data points was 197, 99 and 92 entries for BMI, systolic and diastolic BP, respectively. All analysis was performed using RStudio (RStudio, Inc, Massachusetts, USA; version 3.5.2 released on 20/12/2018). A *p* value < 0.05 was deemed to be statistically significant.

## Results

### Demographics

21,104 patients were randomised into the TIME study. The majority of participants were recruited from England (*n* = 18,532; 87.8%). Remaining participants were from Scotland (*n* = 1816; *n* = 8.6%), Wales (*n* = 750; 3.6%) and Northern Ireland (*n* = 6; 0.03%). The characteristics of participants enrolled in the TIME study are summarised in Table 2. The mean age of all participants at enrolment was 67.7 ± 9.3 years, 12,134 (57.5%) were male, 8892 (42.1%) reported a current or previous history of smoking and diabetes mellitus was the most commonly reported co-morbidity affecting 2764 (13.1%) participants. Mean BMI, derived from self-reported height and weight by 19,593 (92.8%) participants, was 31.6 ± 6.6 kg/m^2^. The mean baseline self-reported systolic and diastolic BPs were 134.9 ± 13.3 mmHg and 78.9 ± 9.4 mmHg, respectively (from 9960 (47.2%) and 9967 (47.2%) participants).

**Table 2:** Characteristics of patients recruited to the TIME study.

	Overall	Do not own an HBPM	Own an HBPM
Total	21,104	9670	11,434
Age (Mean ± SD)	67.7 ± 9.3	67.5 ± 9.8	67.9 ± 8.8
Gender (%)			
Male	12,134 (57.5)	5137 (53.1)	6997 (61.2)
Female	8970 (42.5)	4533 (46.9)	4437 (38.8)
Smoker (%)			
Current	887 (4.2)	500 (5.2)	387 (3.4)
Non-smoker	12,078 (57.2)	5428 (56.1)	6650 (58.1)
Ex-smoker	8005 (37.9)	3662 (37.9)	4343 (38.0)
Unknown	134 (0.6)	80 (0.8)	54 (0.5)
BMI (kg/m^2^) (mean ± SD)		32.2 ± 7.0	31.0 ± 6.0
<30	9116 (43.2)	3771 (39.0)	5345 (46.7)
≥30 (Obese)	10,477 (49.6)	5051 (52.2)	5426 (47.5)
Unknown	1511 (7.2)	848 (8.7)	663 (5.8)
Co-morbidities (%)			
Diabetes	2764 (13.1)	1492 (15.4)	1272 (11.1)
Angina	779 (3.7)	375 (3.9)	404 (3.5)
COPD	615 (2.9)	315 (3.3)	300 (2.6)
Impaired kidney function	681 (3.2)	289 (3.0)	392 (3.4)
Arthritis	1997 (9.5)	945 (9.8)	1052 (9.2)
Peripheral vascular disease	323 (1.5)	175 (1.8)	148 (1.3)
Myocardial Infarction	985 (4.7)	503 (5.2)	482 (4.2)
Stroke/TIA	1239 (5.9)	527 (5.4)	712 (6.2)
Co-morbidity burden (%)			
No co-morbidity	14,147 (67.0)	6283 (65.0)	7864 (68.8)
1–2 co-morbidities	6438 (30.5)	3122 (32.3)	3316 (29.0)
≥3 co-morbidities	519 (2.5)	265 (2.7)	254 (2.2)
Prescribed lipid lowering therapy (%)	7397 (35.1)	3393 (35.1)	4004 (35.0)
Family history (%)			
Parents history of HTN	12,366 (58.6)	5526 (57.1)	6840 (59.8)
Siblings/children HTN history	5942 (28.1)	2623 (27.1)	3319 (29.0)
CV event in 1st degree relative age <60	5049 (23.9)	2342 (24.2)	2707 (23.7)
CV event in 2nd degree relative age <50	1716 (8.1)	833 (8.6)	883 (7.7)
No. of antihypertensive medications (%)			
1	11,845 (56.1)	5681 (58.7)	6164 (53.9)
≥2	8769 (41.6)	3740 (38.7)	5029 (44.0)
Unknown	490 (2.3)	249 (2.6)	241 (2.1)
Socioeconomic deprivation (%)			
More deprived (IMD Decile 1–5)	5953 (28.2)	3080 (31.9)	2873 (25.1)
Less deprived (IMD Decile 6–10)	14,898 (70.6)	6457 (66.8)	8441 (73.8)
Unknown	253 (1.2)	133 (1.4)	120 (1.1)
Country of residence at enrolment (%)			
England	18,532 (87.8)	8277 (85.6)	10,255 (89.7)
Scotland	1816 (8.6)	1004 (10.4)	812 (7.1)
Wales	750 (3.6)	386 (4.0)	364 (3.2)
Northern Ireland	6 (0.0)	3 (0.0)	3 (0.0)
Year of registration to trial			
2011–2013	396 (1.9)	178 (1.9)	218 (1.9)
2014–2015	10,871 (51.5)	5187 (53.6)	5684 (49.7)
2016–2017	9837 (46.6)	4305 (44.5)	5532 (48.4)

*COPD* Chronic obstructive pulmonary disease, *TIA* Transient ischaemic attack, *HTN* hypertension, *IMD* index of multiple deprivation, *CV* Cardiovascular.

### HBPM ownership

11,434 (54.2%) participants reported owning an HBPM, and 10,464 (49.6%) reported the model of HBPM that they owned. In multivariable logistic regression (Table 3), factors significantly associated with a greater likelihood of participants reporting owning an HBPM were: age of 65 and above, male, history of impaired kidney function, history of stroke or TIA, taking two or more antihypertensive medications, parents or siblings/children with a history of hypertension or residence in a less deprived socioeconomic region (IMD 6–10). Participants were significantly less likely to report owning an HBPM if they had a BMI ≥ 30 kg/m^2^, were current smokers, had a history of diabetes mellitus, peripheral vascular disease or MI, were on lipid-lowering therapy, registered for the TIME study in 2014–2015 (compared to in 2016–2017) or residence in Scotland or Wales (compared to England) at study enrolment.

**Table 3:** Logistic regression of factors associated with HBPM ownership amongst participants in the TIME study.

	Univariate analysis		Adjusted analysis	
	OR	95% CI	OR	95% CI
Age				
<65 years	Reference		Reference	
≥65 years	1.15	1.08–1.22	1.1	1.03–1.17
Unknown	0.91	0.70–1.19	0.95	0.73–1.25
Gender				
Female	Reference		Reference	
Male	1.39	1.32–1.47	1.47	1.39–1.56
BMI (kg/m^2^)				
<30	Reference		Reference	
≥30 (Obese)	0.76	0.72–0.80	0.79	0.74–0.84
Unknown	0.55	0.49–0.62	0.64	0.57–0.72
Smoking status				
Non-smoker	Reference		Reference	
Current	0.63	0.55–0.72	0.71	0.62–0.82
Ex-smoker	0.97	0.91–1.02	0.98	0.92–1.04
Unknown	0.55	0.39–0.78	0.68	0.47–0.97
Co-morbidity				
Diabetes mellitus	0.69	0.63–0.74	0.74	0.64–0.86
Angina	0.91	0.79–1.05	1	0.84–1.21
COPD	0.8	0.68–0.94	0.9	0.74–1.09
Impaired kidney function	1.15	0.99–1.35	1.26	1.04–1.52
Arthritis	0.94	0.85–1.03	1.07	0.92–1.24
PVD	0.71	0.57–0.89	0.77	0.60–0.98
Myocardial Infarction	0.8	0.71–0.91	0.82	0.69–0.97
Stroke/TIA	1.15	1.03–1.29	1.23	1.04–1.45
Reported co-morbidity burden				
No co-morbidity	Reference		Reference	
1–2 co-morbidities	0.85	0.80–0.90	0.95	0.82–1.10
≥3 co-morbidities	0.77	0.64–0.91	0.96	0.64–1.42
On lipid lowering therapy	0.86	0.81–0.91	0.84	0.78–0.90
CV event in 1st degree relative age <60	0.96	0.91–1.03	0.99	0.93–1.06
CV event in 2nd degree relative age <50	0.88	0.79–0.97	0.94	0.85–1.04
Parents with history of HTN	1.1	1.04–1.16	1.12	1.05–1.20
Siblings/children with history of HTN	1.08	1.01–1.15	1.1	1.02–1.18
No. of anti-hypertensive medications				
1	Reference		Reference	
≥2	1.24	1.17–1.31	1.25	1.17–1.32
Unknown	0.89	0.74–1.07	0.94	0.77–1.15
Socioeconomic deprivation				
More deprived (IMD Decile 1–5)	Reference		Reference	
Less deprived (IMD Decile 6–10)	1.4	1.32–1.49	1.31	1.23–1.40
Unknown	0.97	0.75–1.24	1.16	0.89–1.51
Country of residence at enrolment				
England	Reference		Reference	
Scotland	0.65	0.59–0.72	0.64	0.58–0.71
Wales	0.76	0.66–1.88	0.74	0.64–0.86
Northern Ireland	0.81	0.15–4.36	0.98	0.18–5.34
Year of registration				
2016–2017	Reference		Reference	
2014–2015	0.85	0.81–0.90	0.84	0.80–0.89
2011–2013	0.95	0.78–1.17	1.07	0.86–1.34

*COPD* Chronic obstructive pulmonary disease, *TIA* transient ischaemic attack, *HTN* hypertension, *IMD* index of multiple deprivation, *CV* cardiovascular.

### Interactions

Male participants with a history of stroke or TIA were found to have a reduced likelihood of owning an HBPM (OR, 0.77; 95% CI, 0.60–0.98). However, no statistically significant interaction was found between gender, other comorbidities and likelihood of owning an HBPM.

## Discussion

### Summary

The results of this study provide practical insights into medical and demographic factors associated with the ownership of HBPMs by patients with hypertension in the UK. This study has identified several factors associated with ownership of HBPMs. Male gender, residence in less socioeconomically deprived areas, taking two or more antihypertensive medications daily, and a history of impaired kidney function or either stroke or TIA were amongst the factors associated with an increased likelihood of owning HBPMs. Conversely, current smokers, participants with a medical history, including diabetes mellitus or peripheral vascular disease or residence in Scotland or Wales (compared to England) at study enrolment were less likely to report owning an HBPM.

### Strengths and limitations

A major strength of this study is that it uses self-reported ownership of HBPM within a very large pragmatic randomised clinical trial. The study population comprises UK adults with diagnosed hypertension, the main target population for HBPM-based interventions. This study does have several limitations. Firstly, it relies solely on patient-reported data, the accuracy of which is not verifiable. Several data fields had erroneous entries which were excluded from subsequent analyses. Patient demographic factors, such as educational attainment and monthly income, which are known predictors of patient engagement with medical devices for self-monitoring, are not collected in the TIME study [19]. This study also relies on data from participants who have opted to be part of an online clinical trial: it is known that clinical trials have an inherent selection bias towards people who are more engaged with their health and, we might assume, would be more likely to have engaged with self-monitoring of their hypertension. The TIME study’s online-only nature may also reduce generalisability as participants were required to have an email address and internet access. It is also unclear whether the statistically significant associations with HBPM ownership observed in this study would remain valid for the wider population, including shift-workers and people taking their antihypertensive medication more than once a day. The general practitioner (GP) role in influencing HBPM ownership was not investigated in this study. The GP-patient relationship, GPs’ medical advice and their perception of the utility of home BP measurement can influence patients’ involvement in self-monitoring of their BP [20].

### Comparison with existing literature

In the TIME study cohort, the rate of HBPM ownership was found to be 54.2%; this is comparable with previously observed HBPM ownership rates which range from 22.8 to 61.7% [11, 19, 21–23]. International differences in patient education on self-monitoring BP and accessibility of HBPMs for private purchase are potential contributing factors to the wide range of HBPM ownership rates observed. Indeed, in less-developed or resource-poor countries, the difficulty of accessing reliable and affordable HBPMs is a recognised challenge [21]. Older age, higher educational status, higher socioeconomic class, non-smoking status, and family history of hypertension are all factors found to be associated with HBPM ownership in previous studies. There is some variation in the influence of gender on HBPM ownership across published literature. However, men have been frequently found to be more likely to own an HBPM, consistent with our findings. Factors associated with poorer health habits, such as smoking and obesity, were associated with a reduced likelihood of owning an HBPM in this study. Although this association seems intuitive, in a survey in Pakistan involving 405 hypertensive adults, those who reported a lack of exercise were, contrastingly, more likely to own HBPMs [11]. The authors of that study, Zahid et al. proposed that those patients, aware of their poorer health habits, were more likely, as a precautionary measure, to self-monitor their BP [11]. It is not known whether issues associated with obesity, such as the need for a larger BP cuff size, which may not come as standard with a machine, or increased discomfort using BP cuffs dissuade patients from acquiring HBPM [24].

Diabetes mellitus, peripheral vascular disease and previous MI were co-morbidities significantly associated with a reduced likelihood of HBPM ownership in this study. In these patient groups, there is evidence of advantages in self-monitoring BP. For example, in people who have a history of MI or diabetes mellitus, self-measured BP readings have been shown to be predictive of complications [25–27]. However, the burden of chronic conditions and associated medications may negatively impact patients’ engagement with their healthcare, possibly explaining lower ownership of HBPMs. This hypothesis is supported by the reduced likelihood of HBPM ownership in patients on additional medication (lipid-lowering drugs) within the TIME study. However, further studies would be required in this subgroup of patients to validate this proposed association [28].

A history of stroke or TIA was associated with an increased likelihood of owning HBPMs in our study. Indeed, in a previous study, a serious health event associated with hypertension, such as stroke, was identified as a motivator for patients to begin self-monitoring BP [21]. However, it must be acknowledged that residual physical disability following a stroke, particularly of the upper limbs, may impair the ability to use HBPMs. Interestingly, men with a history of stroke or TIA were less likely to report owning an HBPM. This association is likely due to proportionately higher engagement rates with HBPM ownership amongst females with a history of stroke or TIA (56.2%, 241/429). In a qualitative study exploring patient experiences of receiving a diagnosis of chronic kidney disease, dialysis was one of the most commonly reported sources of fear [29]. With adequate control of BP delaying the need for renal replacement therapy, this may explain the increased adoption of HBPMs in patients with impaired kidney function observed in this study [30, 31].

### Implications for future research and practice

In this study, ownership of HBPMs was the primary outcome measurement. The frequency of HBPM use (e.g. weekly, monthly) amongst hypertensive patients who own their own devices was not evaluated. Significant variations in frequency of home BP measurement ranging from once daily to less than monthly have previously been reported [1]. The frequency of self-measured BP readings required to observe clinically significant improvement in health outcomes remains unclear. However, a regime of twice each morning and evening for the first week of the month was adopted in the TASMINH4 trial which reported significantly lower BP associated with regular HBPM use. One of the key recommendations in the recent Cross-Party Group report on hypertension in Scotland [32] was to scope the feasibility and cost-effectiveness of the widespread provision of BP monitors to people with high BP and determine the most appropriate mechanism to deliver this. Qualitative research to explore factors which may prevent a patient from using an HBPM regularly and acting upon the results would be valuable to inform any future implementation of BP self-monitoring. Perhaps in the future, HBPM devices will be prescribed for patients.

The prevalence of HBPM ownership in hypertensive patients recruited to the TIME study was approximately half. HBPM ownership among hypertensive patients is increasing; older males, from less deprived socioeconomic regions, with a family history of hypertension, are the demographic most likely to report HBPM ownership. Previous stroke or TIA was associated with an increased likelihood of reporting HBPM ownership within the TIME study cohort. Conversely, chronic conditions such as diabetes mellitus and peripheral vascular disease, and previous MI were associated with a reduced likelihood of HBPM ownership. Further qualitative research would be valuable to identify and explore potential patient-level barriers to engagement with self-monitoring of BP. This may guide prospective evaluation of more targeted interventions to improve ownership and use of HBPMs for self-monitoring of BP.

## Summary

### What is known about topic

- There is increasing evidence that home blood pressure monitoring is an effective strategy to improve BP control in patients with hypertension.
- HBPM ownership prevalence and the factors that influence it are unclear.

### What this study adds

- Within the TIME study cohort, older males, people from less deprived areas, and those with a family history of hypertension, were most likely to report HBPM ownership.
- Chronic conditions such as diabetes mellitus, ischaemic heart disease and peripheral vascular disease, were associated with a reduced likelihood of ownership.
- Recognition of factors associated with HBPM ownership may guide more targeted interventions to promote its use.

## References

[1] McManus RJ, Mant J, Franssen M, Nickless A, Schwartz C, Hodgkinson J, et al. Efficacy of self-monitored blood pressure, with or without telemonitoring, for titration of antihypertensive medication (TASMINH4): an unmasked randomised controlled trial. Lancet. 2018;391:949–59. http://www.ncbi.nlm.nih.gov/pubmed/29499873. 10.1016/S0140-6736(18)30309-X PMC5854463 29499873

[2] McManus RJ, Mant J, Haque MS, Bray EP, Bryan S, Greenfield SM, et al. Effect of self-monitoring and medication self-titration on systolic blood pressure in hypertensive patients at high risk of cardiovascular disease. JAMA. 2014;312:799. http://jama.jamanetwork.com/article.aspx?doi=10.1001/jama.2014.10057. 10.1001/jama.2014.10057 25157723

[3] Sakuma M, Imai Y, Nagai K, Watanabe N, Sakuma H, Minami N, et al. Reproducibility of home blood pressure measurements over a 1-year period. Am J Hypertens. 1997;10:798–803. http://www.ncbi.nlm.nih.gov/pubmed/9234836. 10.1016/s0895-7061(97)00117-9 9234836

[4] Ohkubo T, Imai Y, Tsuji I, Nagai K, Kato J, Kikuchi N, et al. Home blood pressure measurement has a stronger predictive power for mortality than does screening blood pressure measurement: a population-based observation in Ohasama, Japan. J Hypertens. 1998;16:971–5. http://www.ncbi.nlm.nih.gov/pubmed/9794737. 10.1097/00004872-199816070-00010 9794737

[5] Stergiou GS, Bliziotis IA. Home blood pressure monitoring in the diagnosis and treatment of hypertension: a systematic review. Am J Hypertens. 2011;24:123–34. http://www.ncbi.nlm.nih.gov/pubmed/20940712. 10.1038/ajh.2010.194 20940712

[6] George J, MacDonald T. Home blood pressure monitoring. Eur Cardiol. 2015;10:95–101. http://www.ncbi.nlm.nih.gov/pubmed/30310433. 10.15420/ecr.2015.10.2.95 PMC6159400 30310433

[7] Little P, Barnett J, Barnsley L, Marjoram J, Fitzgerald-Barron A, Mant D. Comparison of acceptability of and preferences for different methods of measuring blood pressure in primary care. BMJ. 2002;325:258–9. http://www.ncbi.nlm.nih.gov/pubmed/12153924. 10.1136/bmj.325.7358.258 PMC117641 12153924

[8] Mancia G, Bombelli M, Seravalle G, Grassi G. Diagnosis and management of patients with white-coat and masked hypertension. Nat Rev Cardiol. 2011;8:686–93. http://www.ncbi.nlm.nih.gov/pubmed/21826071. 10.1038/nrcardio.2011.115 21826071

[9] Baral-Grant S, Haque MS, Nouwen A, Greenfield SM, McManus RJ. Self-monitoring of blood pressure in hypertension: a UK primary care survey. Int J Hypertens. 2012;2012:1–4. http://www.ncbi.nlm.nih.gov/pubmed/22013510. 10.1155/2012/582068 PMC3195273 22013510

[10] McManus RJ, Wood S, Bray EP, Glasziou P, Hayen A, Heneghan C, et al. Self-monitoring in hypertension: a web-based survey of primary care physicians. J Hum Hypertens. 2014;28:123–7. http://www.ncbi.nlm.nih.gov/pubmed/23823583. 10.1038/jhh.2013.54 23823583

[11] Zahid H, Amin A, Amin E, Waheed S, Asad A, Faheem A, et al. Prevalence and predictors of use of home sphygmomanometers among hypertensive patients. Cureus. 2017;9:e1155. http://www.ncbi.nlm.nih.gov/pubmed/28503391. 10.7759/cureus.1155 PMC5426820 28503391

[12] Jacob V, Chattopadhyay SK, Proia KK, Hopkins DP, Reynolds J, Thota AB, et al. Economics of self-measured blood pressure monitoring: a community guide systematic review. Am J Prev Med. 2017;53:e105–13. http://www.ncbi.nlm.nih.gov/pubmed/28818277. 10.1016/j.amepre.2017.03.002 PMC5657494 28818277

[13] Uhlig K, Patel K, Ip S, Kitsios GD, Balk EM. Self-measured blood pressure monitoring in the management of hypertension. Ann Intern Med. 2013;159:185. http://www.ncbi.nlm.nih.gov/pubmed/23922064. 10.7326/0003-4819-159-3-201308060-00008 23922064

[14] Grant S, Greenfield SM, Nouwen A, McManus RJ. Improving management and effectiveness of home blood pressure monitoring: a qualitative UK primary care study. Br J Gen Pract. 2015;65:e776–83. http://www.ncbi.nlm.nih.gov/pubmed/26500326. 10.3399/bjgp15X687433 PMC4617273 26500326

[15] Ovaisi S, Ibison J, Leontowitsch M, Cloud G, Oakeshott P, Kerry S. Stroke patients’ perceptions of home blood pressure monitoring: a qualitative study. Br J Gen Pract. 2011;61:e604–10. http://www.ncbi.nlm.nih.gov/pubmed/22152750. 10.3399/bjgp11X593893 PMC3162184 22152750

[16] Rorie DA, Rogers A, Mackenzie IS, Ford I, Webb DJ, Willams B, et al. Methods of a large prospective, randomised, open-label, blinded end-point study comparing morning versus evening dosing in hypertensive patients: the Treatment In Morning versus Evening (TIME) study. BMJ Open. 2016;6:e010313. http://www.ncbi.nlm.nih.gov/pubmed/26861939. 10.1136/bmjopen-2015-010313 PMC4762112 26861939

[17] Stergiou GS, Karpettas N, Atkins N, OʼBrien E. European Society of Hypertension International Protocol for the validation of blood pressure monitors: a critical review of its application and rationale for revision. Blood Press Monit. 2010;15:39–48. http://www.ncbi.nlm.nih.gov/pubmed/20087174. 10.1097/MBP.0b013e3283360eaf 20087174

[18] Lamina C, Sturm G, Kollerits B, Kronenberg F. Visualizing interaction effects: a proposal for presentation and interpretation. J Clin Epidemiol. 2012;65:855–62. https://www.sciencedirect.com/science/article/pii/S0895435612000558. 10.1016/j.jclinepi.2012.02.013 22652348

[19] Akpolat T, Arici M, Sengul S, Derici U, Ulusoy S, Erturk S, et al. Home sphygmomanometers can help in the control of blood pressure: a nationwide field survey. Hypertens Res. 2018;41:460–8. http://www.ncbi.nlm.nih.gov/pubmed/29556094. 10.1038/s41440-018-0030-8 PMC8075910 29556094

[20] Tyson MJ, McElduff P. Self-blood-pressure monitoring—a questionnaire study: response, requirement, training, support-group popularity and recommendations. J Hum Hypertens. 2003;17:51–61. http://www.ncbi.nlm.nih.gov/pubmed/12571617. 10.1038/sj.jhh.1001510 12571617

[21] Viera AJ, Cohen LW, Mitchell CM, Sloane PD. How and why do patients use home blood pressure monitors? Blood Press Monit. 2008;13:133–7. https://insights.ovid.com/crossref?an=00126097-200806000-00001. 10.1097/MBP.0b013e32830263b7 18496286

[22] Tan NC, Khin LW, Pagi R. Home blood-pressure monitoring among hypertensive patients in an Asian population. J Hum Hypertens. 2005;19:559–64. http://www.ncbi.nlm.nih.gov/pubmed/15944723. 10.1038/sj.jhh.1001865 15944723

[23] Akpolat T, Erdem Y, Derici U, Erturk S, Caglar S, Hasanoglu E, et al. Use of home sphygmomanometers in Turkey: a nation-wide survey. Hypertens Res. 2012;35:356–61. http://www.ncbi.nlm.nih.gov/pubmed/22089537. 10.1038/hr.2011.193 22089537

[24] Anast N, Olejniczak M, Ingrande J, Brock-Utne J (2015). The impact of blood pressure cuff location on the accuracy of noninvasive blood pressure measurements in obese patients: an observational study. Can J Anesth.

[25] Kamoi K, Miyakoshi M, Soda S, Kaneko S, Nakagawa O. Usefulness of home blood pressure measurement in the morning in type 2 diabetic patients. Diabetes Care. 2002;25:2218–23. http://www.ncbi.nlm.nih.gov/pubmed/12453964. 10.2337/diacare.25.12.2218 12453964

[26] Oyabu C, Ushigome E, Matsumoto S, Tanaka T, Hasegawa G, Nakamura N, et al. Maximum home blood pressure is a useful indicator of diabetic nephropathy in patients with type 2 diabetes mellitus: KAMOGAWA-HBP study. Diabetes Vasc Dis Res. 2017;14:477–84. http://www.ncbi.nlm.nih.gov/pubmed/28819987. 10.1177/1479164117725477 28819987

[27] Nishimura M, Kato Y, Tanaka T, Taki H, Tone A, Yamada K, et al. Effect of home blood pressure on inducing remission/regression of microalbuminuria in patients with type 2 diabetes mellitus. Am J Hypertens. 2017;30:830–9. http://www.ncbi.nlm.nih.gov/pubmed/28605498. 10.1093/ajh/hpx050 28605498

[28] Matsumoto S, Fukui M, Hamaguchi M, Ushigome E, Matsushita K, Fukuda T, et al. Is home blood pressure reporting in patients with type 2 diabetes reliable? Hypertens Res. 2014;37:741–5. http://www.nature.com/articles/hr201466. 10.1038/hr.2014.66 24718300

[29] Wright Nunes J, Roney M, Kerr E, Ojo A, Fagerlin A. A diagnosis of chronic kidney disease: despite fears patients want to know early. Clin Nephrol. 2016;86:78–86. http://www.ncbi.nlm.nih.gov/pubmed/27345185. 10.5414/CN108831 PMC5012189 27345185

[30] Locatelli F, Del Vecchio L (1999). How long can dialysis be postponed by low protein diet and ACE inhibitors?. Nephrol Dial Transplant.

[31] Klag MJ, Whelton PK, Randall BL, Neaton JD, Brancati FL, Ford CE (1996). Blood pressure and end-stage renal disease in men. N Engl J Med.

[32] Cross-Party Group on Heart Disease and Stroke. Beating High Blood Pressure: Scotland’s Silent Killer. https://www.chss.org.uk/documents/2019/01/beating-high-blood-pressure-scotlands-silent-killer-pdf.pdf. Accessed 17 July 2019.

